# Convulsions during Dentition

**Published:** 1874-02

**Authors:** 


*Convulsions During Dentition, etc.*—J. M. Hall, M. D., in the *Medical and Surgical Reporter*, recommends Valerian in the treatment of this affection, and also gives some general directions to be followed during the continuance of the paroxysm:

"Of late it has been my lot to attend quite a number of little patients affected with convulsions. The patients presented symptoms which were pretty uniform, as follows:

The attack generally commenced in the eyes, which were fixed in one position at first, staring, but as the cases advanced they became agitated, and were turned up beneath the upper eyelids, leaving only the whites visible. The eyelids were sometimes open, sometimes shut; and not unfrequently the eyes were crossed, with pupils dilated or contracted. The muscles of the face next became affected, and the contractions produced at various times violent contortions, the mouth being distorted, sometimes the jaws firmly set, or, again, in violent motion. In a few cases there was foaming at the mouth. In the severer ones, when the spasm became general the whole body was convulsed violently; the head was thrown backward or to either side, the body becoming stiff and rigid, or variously contorted. The fingers and thumbs were drawn into the palms of the hands, the arms thrown backward, or jerked and drawn into all conceivable positions. The lower extremities were likewise sometimes affected, but not generally in so violent a manner. The duration of the "fit" was different in different cases, sometimes continuing for an hour or more, and in the milder cases only lasting for a few minutes.

The most common causes are irritation of bowels from indigestible food, teething, worms, excessive crying and pains, anger and joy. A dangerous form results from overloading the stomach with indigestible food. The most serious case I ever saw to recover was the result of feasting on a large quantity of grapes. The eruptive fevers very frequently produce them. The list of causes might be increased, as I believe the causation may arise from many other sources.

The prognosis in those cases, for the most part, may be favorable, as I believe all will recover except those complicated in some way with cerebral trouble.

The first thing I have done is to prepare a warm bath and put the child in as soon as possible. Where the attacks are very light, a foot bath, with a little mustard, is sufficient; but in the more severe cases, the bath must be a general one. As for medication, I have relied entirely on fluid extract of valerian, in doses varying from ʒ½ to a ʒ, mixed in water and administered as often as fifteen or twenty minutes between the spasms, persistently repeated, with an occasional mild injection of warm water and assafœtida, which gave results so satisfactory that when called to cases of this character I do not feel justified in adopting any other mode of treatment."

